# Supplementary material for: Porous Graphene Oxide Decorated Ion Selective Electrode for Observing Across-Cytomembrane Ion Transport
Source: Sensors (Basel). 2020 Jun 21;20(12):3500. doi: 10.3390/s20123500 (PMC7349088; doi:10.3390/s20123500)
Supplement: Supplementary file 1 [file sensors-20-03500-s001.pdf]

# Supplementary Materials

## Porous Graphene Oxide Decorated Ion Selective Electrode for Observing Across-Cytomembrane Ion Transport


**Shihui Hu, Rong Zhang and Yunfang** Jia *

College of Electronic Information and Optical Engineering, Nankai University, Tianjin 300071, China; 1120170101@mail.nankai.edu.cn (S.H.); 1120190126@mail.nankai.edu.cn (R.Z.)

* Correspondence: jiayf@nankai.edu.cn


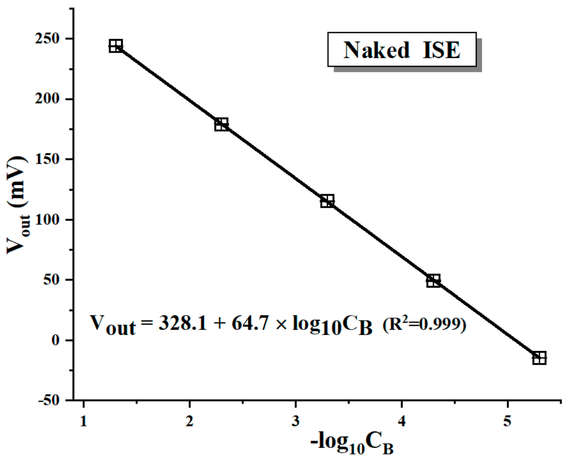


**Figure S1.** Response curve of the naked ISE.

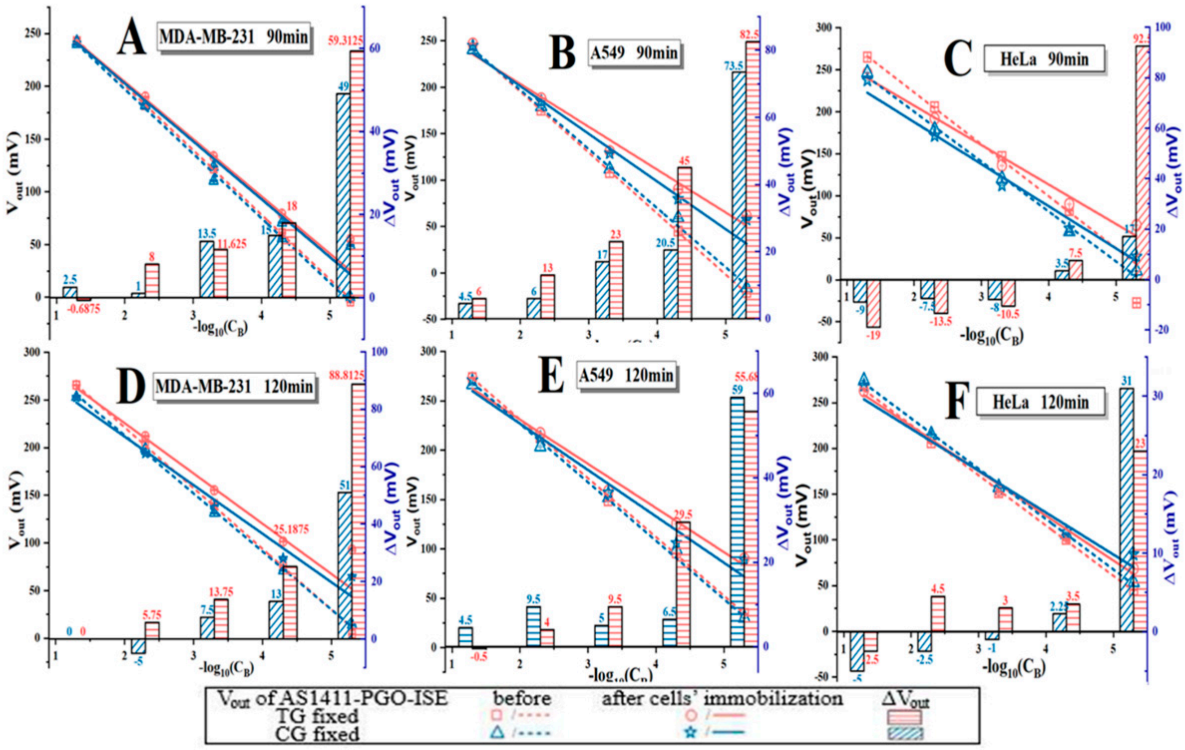

**Figure S2**. Responding curves of the ISEs before and after the immobilization of the cells of MDAMB231 (**A**, **D**), A549 (**B**, **E**) and HeLa (**C**, **F**), which are in the tested group (TG, orange data points and lines) and the control group (CG, blue data points and lines), and suffering the iodide-uptake (IU) operation for 90 (**A-C**) and 120 (**D-F**) min.

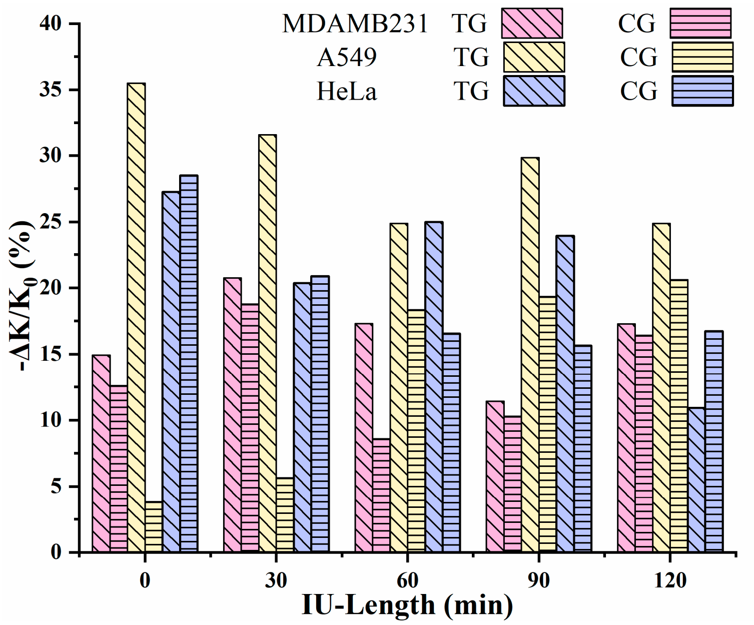


**Figure S3**. The fitting slopes' changing ratios ($-\Delta K/K_0$) of ISE response curves (in **Figure 2** and **Figure S2**) after being fixed by different cells which are MDAMB231 (pink), A549 (yellow) and HeLa (blue). These cells are grouped by with and without NIS expression on their cytomembranes, named as TG and CG, respectively. Meanwhile, these cells are also varied by whether they have suffered the iodide uptake (IU) treatment, and the lengths of IU (0, 30, 60, 90, 120 min). For the columns in the same color, the higher ones are always found by the TG ones which are the cells treated by IU, except for the cells of HeLa (blue) with IU length of 0, 30 and 120 min. The increased columns by most of TG cells indicate the sensitivities of cells fixed ISEs are lowered more by the TG cells than the CG ones, the reason may be that the ion concentrations in the IME ($C_I$) are probably increased by the NIS facilitated ion transport and the osmosis of $Cl^-$, as mentioned in the manuscript. However, we could not find obvious deviations among the columns of different IU lengths.
